# Language Interfaces in Adult Heritage Language Acquisition: A Study on Encoding of Nominal Reference in Mandarin Chinese as a Heritage Language

**DOI:** 10.3389/fpsyg.2021.790102

**Published:** 2022-01-04

**Authors:** Jing Jin, Sihui Echo Ke, John Chi-Kin Lee

**Affiliations:** ^1^Department of Chinese Language Studies, The Education University of Hong Kong, Hong Kong, Hong Kong SAR, China; ^2^Department of Modern and Classical Languages, Literatures and Cultures, University of Kentucky, Lexington, KY, United States; ^3^Department of Curriculum and Instruction, The Education University of Hong Kong, Hong Kong, Hong Kong SAR, China

**Keywords:** heritage language, Interface Hypothesis, syntax-semantics/syntax-discourse interface, nominal reference, Mandarin Chinese

## Abstract

According to the Interface Hypothesis in the field of bilingualism, the interface connecting a linguistic module with a language-external domain (e.g., syntax-discourse) will present prolonged difficulties for adult bilingual learners, as compared with the interface connecting language-internal modules (e.g., syntax-semantics). This study tested whether the Interface Hypothesis is applicable to the acquisition of Mandarin Chinese as a heritage language. An internet-based acceptability judgment task (AJT) was administered to 58 advanced and intermediate adult Chinese heritage speakers to collect data in accuracy and reaction time to investigate the adult heritage speakers’ mastery of referential nominal expressions regulated at the syntax-semantics and syntax-discourse interfaces, respectively, in Mandarin Chinese. The target linguistic phenomena involved three nominal expressions (i.e., the bare N(oun), the [Cl(assifier)-N], and the [Num(eral)-Cl-N]) under four interface-regulated referential readings (i.e., type-denoting, quantity-denoting, indefinite individual-denoting, and definite individual-denoting). In terms of accuracy, the results showed that (i) for the N and the [Num-Cl-N], regardless of the interface type, the advanced group acquired the target phenomena to a nativelike level, who significantly outperformed the intermediate group; (ii) for the [Cl-N], the advanced group exhibited nativelike attainment at the syntax-discourse interface but not at the syntax-semantics interface, and performed significantly better than the intermediate group at both interfaces. Regarding reaction time, no significant differences were reported between the advanced group and the native group for the target structures at either the syntax-semantics or the syntax-discourse interface, while the advanced group performed significantly better than the intermediate group, regardless of the interface type and the structure type. The findings suggest that the nature of the language interface, i.e., whether it pertains to language-external domains (i.e., the external interface) or not (i.e., the internal interface), should not be a reliable factor for predicting the (im)possibility of nativelike attainment of bilingual grammar knowledge, contra the predictions of the Interface Hypothesis. The present study provides new empirical evidence to show that language-external interface properties are not necessarily destined for prolonged difficulties in heritage language acquisition, and that it is possible for adult heritage speakers to make developmental progress in both accuracy and processing efficiency at different types of interfaces.

## Introduction

In the field of bilingualism research under the generative linguistic framework, an issue that has been of immense scholarly interest in the past decade is the discrepancy of learning difficulties exhibited by different linguistic modules. To account for the patterns of non-convergence and residual optionality shown by bilinguals, an influential hypothesis, i.e., the Interface Hypothesis (IH hereafter), is proposed in the literature. The IH was first put forth to explain the non-nativelike attainment at the end stage of adult second language (L2) acquisition, which claimed that the language structures involving an interface between syntax and other domains would exhibit persistent vulnerability as compared with those involving purely syntactic properties (i.e., the so-called “narrow syntax”) (Sorace, 2000, 2005; Sorace and Filiaci, 2006; Belletti et al., 2007). A later version of the IH makes an internal vs. external interface distinction, predicting that adult L2 learners may eventually achieve nativelike acquisition at the internal interface, i.e., the interface connecting language-internal modules, such as syntax-semantics, whereas there will be prolonged optionality for adult L2 learners at the external interface, i.e., the interface connecting a linguistic module with a language-external domain, such as syntax-discourse (Tsimpli and Sorace, 2006; Sorace and Serratrice, 2009; White, 2011).^1^

In addition to the advanced stage of adult L2 acquisition, the IH has also been extended to early bilingual first language (L1) acquisition and the early stage of L1 attrition [See Sorace (2011) and the references therein], and later to heritage language acquisition (e.g., Lardiere, 2011; Montrul and Polinsky, 2011; White, 2011).^2^ As such, currently, the IH provides a unifying framework for bilingual language acquisition. While the IH has generated a fruitful body of empirical research in the field of bilingualism, the results obtained so far were highly mixed, with some studies verifying the IH whereas others not, no matter for the much-studied area of L2 acquisition at interfaces or for the lately emerging area concerning heritage language acquisition at interfaces. The present study will contribute to the ongoing debate on the IH *via* presenting new evidence from the perspective of adult heritage speakers’ acquisition of interface-regulated referential nominal expressions in Mandarin Chinese as a heritage language, which remains an under-explored area in the prior studies.

To begin with, a brief introduction to the definitions of “heritage speakers” and “heritage languages” is warranted. By “heritage speakers,” it means bilingual speakers who grow up in an asymmetrical bilingual environment where the language spoken at home, i.e., the heritage language, is not the dominant language of the society, i.e., the societal language (Montrul, 2008, 2016; Rothman, 2009). Heritage speakers are early bilinguals as they are exposed to both the heritage language and the societal language since their birth or in childhood. As heritage speakers “have been raised with a strong cultural connection to a particular language through family interaction” (Van Deusen-Scholl, 2003, 222), the heritage language is commonly perceived as representing familial, cultural, and ancestry ties of heritage speakers (Berardi-Wiltshire, 2018). However, heritage speakers may undergo a shift in linguistic dominance and ultimately exhibit a stronger command of the societal language—with the heritage language as a weaker language—by the time they reach adulthood as a result of a much wider adoption of the societal language for purposes of education, work, daily social, etc. As indicated by many studies, heritage speakers’ competence in the language *L* as a heritage language tends to be different, both quantitatively and qualitatively, from that of monolingual speakers of *L* as a native language, and heritage language grammars display representational and processing differences from monolingual grammars [see Montrul (2012, 2016) and the references therein].

Given the extensive scope and scale of Chinese people’s migration and mobility all over the world nowadays, and the changing perceptions about the utility and importance of the Chinese language (He, 2006; Duff et al., 2017), there are of both theoretical and pedagogical values to extend the scope of research on interface grammar to Chinese heritage speakers, an under-examined population in the literature on the IH. As indicated in a review by Ma et al. (2017), researchers and teachers may draw on the similarities and differences between Chinese heritage speakers and non-Chinese heritage speakers in order to understand what the two kinds of learners have in common and how they differ. The present study is aimed to investigate adult Chinese heritage speakers’ mastery of interface-regulated referential nominal expressions, which remains an under-explored area in the prior studies. The study will advance the current discussion on heritage language acquisition at interfaces in three dimensions: (i) target language: while most of the prior research targeted Indo-European heritage languages, this study extends the scope of exploration to an under-researched heritage language, i.e., Mandarin Chinese as a heritage language; (ii) target phenomenon: while the target phenomena of the existing studies were mostly concerned with the external interface only, the present study features a comparison of heritage speakers’ mastery of internal interface and external interface grammar knowledge in the heritage language; and (iii) methods: while the instruments adopted in previous studies were mainly restricted to offline tasks, this study evaluates heritage speakers’ performance at interfaces *via* real-time paradigms examining both accuracy and processing proficiency.

The content of this paper is organized as follows. Section “Previous Studies: Bilingual Acquisition at Interfaces” briefly reviews previous studies examining bilingual acquisition at interfaces, particularly heritage language acquisition at interfaces; Section “Linguistic Phenomenon: Referentiality Encoding of Chinese Nominals” provides a brief description of the linguistic phenomenon targeted by the present study; Section “The Present Study” presents the “Research Questions and Hypotheses,” “Methods,” “Results,” “Discussion,” and “Conclusion” of the present work.

## Previous Studies: Bilingual Acquisition at Interfaces

With the increase of empirical investigations testing the IH with different populations *via* different tasks, it has been noted that bilingual learners’ mastery of internal vs. external interface knowledge exhibits a quite complicated picture. This section will provide a brief review of previous studies on the IH, with a special focus on heritage language acquisition at interfaces.

As the IH was originally proposed to account for the end stage of adult L2 acquisition at interfaces, so far, L2 acquisition at interfaces has received particularly considerable attention. The results reported in the literature were mixed: while some studies showed that advanced L2 learners could achieve nativelike attainment only at the internal interface but not at the external interface, which borne out the IH (e.g., Lozano, 2006; Valenzuela, 2006; Belletti et al., 2007), others found that advance L2 learners could also master the external interface knowledge to a nativelike level, which constituted evidence against the IH (e.g., Donaldson, 2012; Ivanov, 2012; Leal, 2016). Moreover, it was observed that other variables such as L1 background and task design may also affect the results of the experiments on interface grammar acquisition in that, differences in these variables might lead to inconsistent observations regarding whether L2 learners’ performance at interfaces would be comparable to that of the native speakers’ (Hopp, 2007; Slabakova and Ivanov, 2011). Recently, the processing dimension regarding interface grammar has attracted growing interest among scholars (e.g., Sorace and Serratrice, 2009; Wilson, 2009; Slabakova and Ivanov, 2011; Laleko and Polinsky, 2016; Leal et al., 2017). The latest version of the IH has explicitly attributed non-convergence and residual optionality at the external interface to a greater processing burden for interfacing linguistic modules with language-external domains (Sorace, 2011). Such perspective is, nevertheless, not without controversy. For instance, regarding adult L2 Chinese acquisition of the interface-regulated word order rules of prenominal modifiers in the numeral classifier construction as demonstrated above in footnote 1, the reaction time data from the advanced L2 Chinese speakers did not report significant differences between the word order phenomenon conditioned at the syntax-semantics interface and that conditioned at the syntax-discourse interface, which showed that the internal vs. external interface distinction did not suffice to determine whether a given interface grammar property (e.g., an interface-regulated rule on word ordering) would require more (or less) processing efforts for adult L2 learners (e.g., Jin and Ke, 2021).

Besides L2 acquisition, empirical investigations on the IH have also been extended to heritage speakers’ acquisition of interface grammar properties. Similar to the cases of L2 acquisition at interfaces, inconsistent results have been reported regarding heritage language acquisition at interfaces. On the one hand, there are studies providing evidence to support the IH. For example, Keating et al. (2011) adopted an offline questionnaire to examine adult Spanish heritage speakers’ antecedent preferences for null and overt pronouns in ambiguous complex sentences that consist of a main clause followed by a subordinate clause, a syntax-discourse interface phenomenon in Spanish. It was found that the resolution of intrasentential anaphora was a locus of instability for the heritage speakers, a result well bearing out the prediction of the IH. Pascual y Cabo et al. (2012) used an offline scalar judgment felicitousness task to probe into adult Spanish heritage speakers’ knowledge of non-obligatory subjunctive mood as complements of epistemic predicates, which is also a syntax-discourse interface phenomenon in Spanish. Data showed that while the heritage speakers exhibited full competence of the syntax of volitional subjunctive, they exhibited non-nativelike performance in modality selection (indicative vs. subjunctive) for complements of epistemic predicates, which confirmed the prediction of the IH.

On the other hand, there are a considerable number of studies on heritage language acquisition arguing against the IH. Most of these studies examined Spanish as a heritage language, with the target phenomena including the use of definite articles (Montrul and Ionin, 2010), subject position preferences with intransitive predicates across informational contexts (De Prada Pérez and Pascual y Cabo, 2012), clitic right dislocation (Leal et al., 2014), presentational focus (Hoot, 2017), etc., all of which are regulated at the external interface in Spanish. A few studies addressed acquisition at interfaces in East Asian languages as heritage languages, with the target phenomena examined so far including topic markers in heritage Korean and Japanese (Laleko and Polinsky, 2016) and null objects in heritage Mandarin Chinese (Chou et al., 2020).^3^ In terms of methods, these studies all adopted acceptability/felicity judgment tasks as the main instruments (although the specific design of each study may vary). The results showed that heritage language acquisition at the external interface was more complicated than was assumed under the IH and could not be simply attributed to generalized interface-related deficits.

Meanwhile, there are studies partially confirming the predictions of the IH. For example, Yan (2020) adopted a set of instruments including the acceptability judgment task (AJT), the dialog completion task, and the translation task to investigate adult Chinese heritage speakers’ mastery of the syntactic and discourse features of the sentence final particle *ba* in Chinese. The results showed that while the “suggestion” discourse feature of *ba* imposed prolonged difficulties for the heritage speakers, which was consistent with the IH, the “question” discourse feature of *ba* could be eventually acquired to a nativelike level, which contradicted the IH. This suggested that the claimed vulnerability in the syntax-discourse domain for heritage speakers (e.g., Montrul, 2012) may not be applicable across the board.

Albeit various attempts have been made to test the IH with heritage speakers in the literature, the existing empirical investigations are far from conclusive. To be specific, there are three main research niches. First, the target heritage languages in previous studies were highly limited. As reviewed above, most of the prior research targeted Indo-European heritage languages (particularly Spanish), whereas East Asian languages were notably under-explored. Second, the target phenomena of the existing studies mostly pertained to the external interface only. For a more precise understanding about the predictability of the IH, studies featuring a comparison of heritage language acquisition at the internal interface vs. the external interface are called for. Third, the experimental instruments adopted were mainly restricted to offline tasks. Compared with the research on L2 acquisition at interfaces, there is an evident scarcity of research adopting online tasks to scrutinize the real-time processing of heritage speakers’ interface grammar knowledge.

To fill the above gaps, the present study will investigate adult learners’ performance of referential nominal expressions regulated at internal vs. external interfaces in Mandarin Chinese as a heritage language. The present study is aimed to advance the current discussion on heritage language acquisition at interfaces in three aspects: (i) to extend the scope of exploration into the under-researched heritage language (i.e., Chinese), (ii) to conduct a comparison of heritage speakers’ mastery of internal vs. external interface grammar in the heritage language, and (iii) to examine heritage speakers’ performance at interfaces in both accuracy and processing proficiency.

## Linguistic Phenomenon: Referentiality Encoding of Chinese Nominals

The target linguistic phenomenon of the present study concerns encoding of different referential meanings by nominal expressions in Chinese. The present study targets this phenomenon because referential nominal expressions are essential building blocks of languages for making reference, the correct interpretation and appropriate use of which are crucial for ensuring smooth communication. The referential meanings tested include four types: (i) the type-denoting reading, i.e., the nominal refers to an entity type, (ii) the definite individual-denoting reading, i.e., the nominal refers to the contextual discourse referent(s) simultaneously identifiable to both the speaker and the hearer, (iii) the indefinite individual-denoting reading, i.e., the nominal refers to the referent(s) only contextually identifiable to the speaker but not to the hearer, and (iv) the non-referential, quantity-denoting reading, i.e., the nominal expresses the amount/number of something (cf. Heim, 1982).

The nominal structures examined include the following three types:

(I) Bare N(oun)s. At the lexical semantic level, Chinese bare Ns denote a type meaning, as shown in (1a); while in certain contexts, they could also be used as indefinite or definite individual-denoting expressions, as given in (1b) and (1c), respectively (e.g., Chierchia, 1998; Liao and Wang, 2011; Jin, 2013). When under the individual-denoting usage, Ns are compatible with either plural or singular readings, depending on the context in which they are uttered. The quantity-denoting meaning is not available for bare Ns.


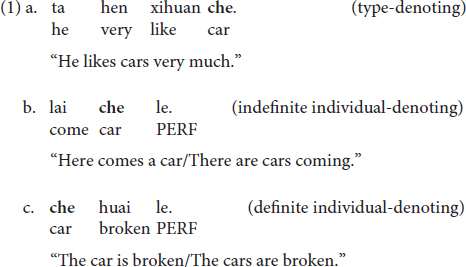


(II) [C(lassifier)-N(oun)]. [Cl-N] can only be used as an indefinite individual-denoting expression in Mandarin Chinese (Cheng and Sybesma, 1999; Jin, 2013), as shown in (2). Type-denoting, definite individual-denoting, and quantity-denoting readings are all unavailable for [Cl-N].


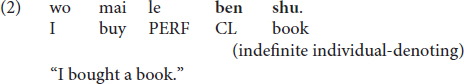


(III) [Num(eral)-Cl-N]. The [Num-Cl-N] sequence in Mandarin Chinese is compatible with two uses, one as an indefinite individual-denoting expression, under which it is associated with some existential referent(s), the other as a quantity-denoting expression, under which it is for cardinality counting purposes (Li, 1998), as given in (3a) and (3b), respectively. Neither the definite individual-denoting nor the type-denoting reading is available for [Num-Cl-N] (Cheng and Sybesma, 1999).


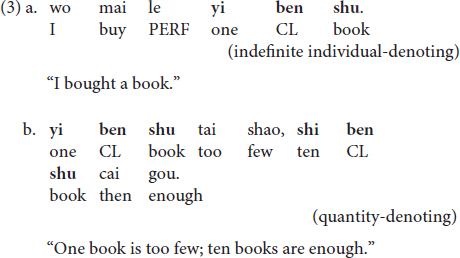


The various uses of the three expressions represent a case of complex interface phenomenon in Mandarin Chinese. To be concrete, for bare Ns, while their use as a type-denoting expression is lexically semantics-regulated (Chierchia, 1998; Liao and Wang, 2011; Jin, 2013, 2018), the definite and indefinite individual-denoting uses are determined at the discourse level, regulated by factors such as context, the cognitive status of interlocutors, the co-occurring predicates, etc. (Li and Thompson, 1981; Simpson et al., 2011). For [Num-Cl-N], the quantity-denoting meaning is determined at the lexical semantic level due to the existence of the numeral, while the indefinite individual-denoting meaning is introduced at the discourse level when the quantity has been contextually associated with existential referents (Li, 1998). For [Cl-N], due to the absence of the numeral, the quantity-denoting use is inherently unavailable at the semantic level (Jin, 2013); however, its use as an indefinite individual-denoting expression can be licensed at the discourse level when a referential relationship has been contextually established between [Cl-N] and an existential referent. Based on the internal vs. external interface distinction assumed under the IH, depending on whether the referential meanings encoded by the structures are licensed at the discourse level (i.e., the external interface) or at the lexical-semantic level (i.e., the internal interface), the various uses of the three nominal expressions can fall under the interface subcategorization as summarized below:

(4) Interfaces associated with the uses of Ns, [Cl-N], and [Num-Cl-N] in Mandarin Chinese^4^


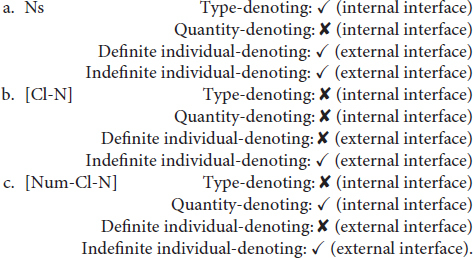


## The Present Study

### Research Questions and Hypotheses

The present study is guided by the following three research questions (RQs): (1) Can advanced adult Chinese heritage speakers master the syntax-semantics and syntax-discourse interface knowledge of referential expressions to a nativelike level? (2) Does the overall Chinese proficiency affect adult Chinese heritage speakers’ mastery of the target referential expressions at the syntax-semantics and syntax-discourse interfaces? (3) Is the heritage speakers’ acquisition of the target interface-regulated referential expressions mediated by syntactic structure complexity?

In the spirit of the IH, the present study formulates the following hypotheses: (1) the advanced adult heritage speakers can achieve target-like attainment of the target expressions at the internal interface but not at the external interface; (2) the heritage speakers’ performance at the syntax-semantics interface, but not at the syntax-discourse interface, is positively related to the heritage language proficiency level; (3) the more complex the syntactic structure is, the more difficult for the heritage speakers to master the interface constraints on the use of the structure.

### Methods

#### Participants

The study recruited 58 adult heritage speakers of Chinese coming from overseas and 29 native speakers of Chinese. The heritage speakers, 42 female and 16 male, were students of four universities in mainland China enrolled in a variety of programs, including Chinese as a second language, international trade, business administration, computer science, communication, food science, architecture, etc. At the time of the study, their age ranged from 18 to 28. They came from 11 nations, including six Spanish-speaking countries (Venezuela, *n* = 14; Peru, *n* = 9; Panama, *n* = 3; Columbia, *n* = 3; Bolivia, *n* = 1; Ecuador, *n* = 1), two English-speaking countries (Canada, *n* = 3; Australia, *n* = 3), as well as Malaysia (*n* = 18), Brazil (*n* = 2), and Indonesia (*n* = 1), each with its own official language. Given that initial exposure to the heritage language—which in turn subsumes the important factors such as age of acquisition, nature/timing of input, etc.—is crucial to heritage speakers’ mastery of this language (Montrul, 2010a,^2012^), the heritage speakers under the present study were divided into an advanced group (*n* = 29) and an intermediate group (*n* = 29) according to the age at which they were initially exposed to Mandarin Chinese learning at school, a classification which was in the meanwhile validated against the heritage speakers’ self-rating of their own overall Chinese proficiency. To be specific, those who started learning Mandarin Chinese in primary school were designated as the advanced group, while those who started in secondary school or college were designated as the intermediate group, i.e., the initial exposure to Mandarin Chinese provided a natural boundary between the two groups of heritage learners, and served as a proxy measure of Chinese proficiency. To cross-validate this measure, the heritage learners were asked to rate their own overall Chinese proficiency (including listening, reading, and speaking) on three 10-point Likert scales.^5^ The resulting composite scores (alpha = 0.916) of the two groups were compared through an independent-samples *t*-test (one-tailed), according to which the advanced group (*M* = 25.93, *sd* = 4.15) had significantly higher proficiency than the intermediate group (*M* = 18.64, *sd* = 4.08), *t* = 6.69, *p* < 0.001.

The 29 native speakers of Chinese, 28 female and one male, were all undergraduate and post-graduate students from two universities in mainland China, aged between 17 and 32 at the time of the study. They were all natives of the northern provinces of mainland China, where dialects of Mandarin Chinese are spoken.

#### Instruments

An internet-based AJT was administered to all the participants. The task comprised 84 stimuli, of which 24 involved bare Ns, 24 the [Cl-N] structure, and 24 the [Num-Cl-N] structure. Each category consisted of 12 internal interface-regulated items and 12 external interface-regulated items (please refer to see section “Appendix: Sample Items of the Online Acceptability Judgment Task” for examples). Each set of 12 items made up a distinct scale in subsequent data analysis. Also included were 12 fillers irrelevant to the present study. For each item, the participants were given a hypothetical context followed by a short question and an answer containing the target expression. The participants were asked to judge in the shortest possible time whether the presented expression was acceptable or not by pressing the relevant keys on the computer keyboard; if they did not understand a certain stimulus, they were allowed to choose the “I don’t know” option (Hopp, 2005). The 84 stimuli were presented in random order so that the order of presentation was different across participants. Both their responses and reaction times were recorded.

A typical item was presented in two steps. First, the context was presented in written form on the computer screen (in Chinese only), as exemplified in (5). For ease of the participants’ understanding, the hypothetical contexts adopted in the AJT were all closely related to situations commonly experienced in everyday life (e.g., eating, shopping, cooking, etc.).

(5) A和B在讨论食物。 (“A and B are talking about food.”).

No time limit was imposed on the context instruction (Taguchi, 2007). After reading, the participants could press any key on the keyboard to proceed to step two, when a question-and-answer conversation between speakers A and B were presented on the screen in written form (in Chinese only). For example:

(6) A: 你喜欢吃什么？ (“What do you like eating?”).
  B: 我喜欢吃个西红柿。 (“I like eating a tomato.”).

The participants were asked to judge whether speaker B’s answer is correct or not without time constraints (Taguchi, 2005, 2007) according to the context and speaker A’s question. They pressed the F-key for a correct answer, the J-key for an incorrect answer, and the spacebar if they were unsure. After the participants made a judgment, the test proceeded to the next item automatically.

#### Data Collection Procedures

The internet-based AJT was administered to each of the participants one by one *via* the Gorilla online platform (Anwyl-Irvine et al., 2020). Prior to the AJT, the participants were required to ascertain that they had sufficient time to complete the task in a single trial and that they had good access to internet. As reaction time data were to be analyzed, they were also required to use a desk-top or lap-top with a standard physical keyboard, instead of a tablet or smartphone, so as to minimize the effect of hardware on their reaction time. The participants also signed an informed consent form online prior to the AJT. As the participants were all new to the online platform, and social distancing due to COVID-19 prevented physical contacts, three measures were taken to make sure the right procedures were followed by the participants. First, the online directions were presented in two languages (Chinese and English) and proofread by the first four participants to make sure they understood the procedures. Second, the directions were sent to the participants *via* an instant messaging platform with one example item, so that they could request clarification should they have any doubt. Third, a practice session with four extra items was administered prior to the AJT, and the participants were encouraged to seek help from the administrator on the instant messaging platform should they encounter any problems. Most participants were able to complete the AJT within 30 min.

The Gorilla platform automatically recorded the responses and the reaction times, which were downloaded as a Microsoft Excel file. Despite the aforementioned efforts, data screening discovered a few irregularities, including unexpectedly short reaction times (within one second for many items), interrupted trials (with item reaction times that lasted more than 30 s), and patterned responses, such as the same response for all items. Furthermore, a few participants were found to be disqualified as participants, either because they were born and raised in China in their early years, or because they were not studying in Chinese universities, which may bring in construct-irrelevant variance (Messick, 1995). Originally 94 participants were invited, but after deleting the cases of irregularities, the data from 87 participants (including both heritage and native speakers) were retained, whose demographic details were reported above.

#### Data Analyses

The response dataset included the mean accuracy rate and the mean reaction time of each participant on each 12-item scale featuring a distinct intersection between interface (internal vs. external) and structure (bare Ns, [Cl-N], and [Num-Cl-N]). The “I don’t know” responses were excluded from the calculation of these mean values. Of the 87 (participants) × 6 (scales) = 522 mean values of either accuracy rate or reaction time, only one was calculated with six “I don’t know” responses, one with five unsure responses, five with four unsure responses, and eight with three unsure responses. Due to the scarcity of the “I don’t know” responses, the effect of excluding them from the calculation of mean values on the reliability of the scales was considered negligible [see also Hopp (2005)].

The internal consistency reliability was estimated for each 12-item scale. As Table 1 shows, the coefficient alpha estimates ranged between 0.705 and 0.904, and were considered sufficient for subsequent analyses.

**TABLE 1:** Coefficient alpha estimates by scale.

Structure	Number of items	Acceptability judgment task		Reaction time	
		Internal	External	Internal	External
Bare N	12	0.713	0.705	0.751	0.887
Cl-N	12	0.899	0.708	0.903	0.904
Num-Cl-N	12	0.817	0.763	0.886	0.876

Repeated-measures ANOVAs were conducted in SPSS 24 to answer the research questions, treating the mean accuracy rate of each participant as the dependent variable in one series, and the mean reaction time of each participant in the other. In Model 1 of each series, interface was entered as the within-subject factor, participant group (native, advanced, and intermediate) as the between-subject factor, and the group × interface interaction effect was estimated to answer RQ1 and RQ2. In Model 2 of each series, syntactic structure was added as another within-subject factor, and the three-way interaction (group × interface × structure) was estimated to answer RQ3.

### Results

#### Descriptive Statistics

**FIGURE 1:**
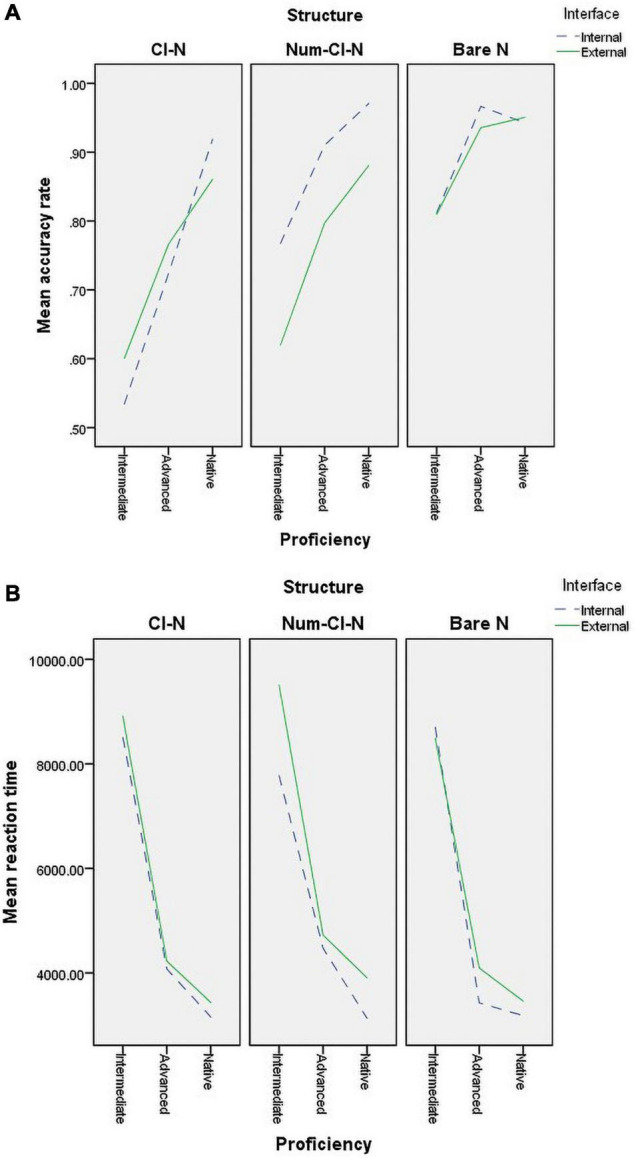
Mean accuracy rate and reaction time by group, interface, and structure. **(A)** Mean accuracy rate. **(B)** Mean reaction time.

Table 2 shows the mean accuracy rate and reaction time and the corresponding standard deviation for each group in each interface, and each interface-structure combination. The means are also graphically presented in Figure 1.

**TABLE 2:** Descriptive statistics of accuracy rate and reaction time by group, interface and syntactic structure.

Structure	Proficiency	*n*	Accuracy rate mean (sd) in%		Reaction time mean (sd) in msec.	
			Internal	External	Internal	External
Bare N	Intermediate	29	81.20 (17.92)	80.97 (17.28)	8695.73 (6698.94)	8483.29 (3338.28)
	Advanced	29	96.64 (7.44)	93.55 (8.38)	3426.20 (1788.91)	4092.54 (2267.07)
	Native	29	94.25 (7.08)	95.06 (7.32)	3180.37 (1132.69)	3459.97 (1247.06)
	All	87	90.70 (13.62)	89.86 (13.34)	5100.77 (4755.79)	5345.27 (3294.89)
Cl-N	Intermediate	29	53.43 (26.96)	60.06 (16.18)	8497.31 (2888.19)	8909.44 (3399.61)
	Advanced	29	72.35 (25.98)	76.61 (16.74)	4073.55 (1711.95)	4224.96 (2336.38)
	Native	29	91.88 (14.10)	86.05 (14.23)	3155.59 (939.15)	3433.58 (1138.42)
	All	87	72.55 (27.76)	74.24 (18.95)	5242.15 (3075.55)	5522.66 (3445.21)
Num-Cl-N	Intermediate	29	76.75 (18.78)	61.99 (15.58)	7774.36 (3046.84)	9504.40 (3847.66)
	Advanced	29	91.01 (13.90)	79.72 (17.83)	4470.85 (2863.57)	4725.14 (2618.78)
	Native	29	97.07 (9.56)	88.06 (15.94)	3132.14 (860.19)	3904.00 (1131.95)
	All	87	88.28 (16.76)	76.59 (19.62)	5125.78 (3127.87)	6044.51 (3693.07)
Total	Intermediate	87	70.46 (24.62)	67.68 (18.75)	8322.47 (4528.54)	8965.71 (3519.75)
	Advanced	87	86.67 (20.24)	83.30 (16.51)	3990.20 (2203.05)	4347.55 (2399.69)
	Native	87	94.40 (10.74)	89.72 (13.46)	3156.03 (972.72)	3599.18 (1180.07)
	All	261	83.84 (21.77)	80.23 (18.77)	5156.23 (3721.54)	5637.48 (3480.91)

Figure 1 provides some initial information for planning the statistical modeling. As Figure 1A shows, there was some group × interface interaction in mean accuracy rate, but only in the case of the [Cl-N] structure, not for the other two structures. This was taken as a sign of three-way interaction. In Figure 1B, however, there seemed to be no apparent group × interface interaction in mean reaction time, regardless of structure.

#### Interface Knowledge of Referential Expressions Among Adult Native Speakers, Adult Advanced Heritage Speakers, and Adult Intermediate Heritage Speakers

To reiterate, to answer RQ1 and RQ2, interface was treated as a within-subject factor and group as a between-subject factor. As the within-subject factor had only two levels, the sphericity assumption was automatically sustained. In the case of accuracy rate, the two-way repeated-measures ANOVA (group × interface) discovered a significant main effect for group, *F*_(2, 84)_ = 30.810, *p* < 0.01, η^2^ = 0.423, and a significant main effect for interface, *F*_(1, 84)_ = 8.775, *p* < 0.01, η^2^ = 0.095, but no significant two-way interaction was found, *F*_(2, 84)_ = 0.210, *p* > 0.05, η^2^ = 0.005. Pairwise comparisons were conducted to identify the specific differences between the groups.

As shown in Table 3, the mean accuracy rate of the advanced group was significantly lower than that of the native group, and the mean accuracy rate of the intermediate group was significantly lower than that of the advanced group, regardless of interface type. The omnibus test yielded similar results for reaction time, with a significant main effect for group, *F*_(2, 84)_ = 51.188, *p* < 0.01, η^2^ = 0.549, and a significant main effect for interface, *F*_(1, 84)_ = 8.063, *p* < 0.01, η^2^ = 0.088, but no significant two-way interaction, *F*_(2, 84)_ = 0.250, *p* > 0.05, η^2^ = 0.006. The mean reaction time of the advanced group was not significantly longer than that of the native group, but the mean reaction time of the intermediate group was significantly longer than those of the advanced and native groups, regardless of interface type.

**TABLE 3:** Pairwise comparison results for the two-way interactions.

Measure	Interface	Intermediate vs. Advanced		Intermediate vs. Native		Advanced vs. Native	
		Mean difference	SE	Mean difference	SE	Mean difference	SE
Accuracy rate	Internal	−16.21**	3.73	−23.94**	3.73	−7.73*	3.73
	External	−15.62**	2.93	−22.05**	2.93	−6.43*	2.93
Reaction time	Internal	4332.27**	600.77	5166.43**	600.77	834.17	600.77
	External	4618.16**	596.21	5366.53**	596.21	748.37	596.21

*p < 0.05; **p < 0.01.

In sum, to answer RQ1, nativelike attainment was observed in reaction times in advanced heritage speakers, but not in their accuracy rates. In response to RQ2, there was a significant developmental progress in both accuracy and reaction times as proficiency levels increased from the intermediate to the advanced level among heritage language speakers.

#### Effect of Syntactic Structure Complexity

To answer RQ3, repeated-measures ANOVA incorporated syntactic structure as an additional within-subject factor. Mauchly’s test discovered some violations of the sphericity assumption, *W* = 0.662, *p* < 0.01 for the structure factor in the accuracy rate model, *W* = 0.755, *p* < 0.01 for the structure factor, and *W* = 0.482, *p* < 0.01 for the interface structure interaction in the reaction time model. Therefore, results based on the Greenhouse-Geisser correction were reported where applicable. The key to RQ3 is the three-way interaction between group, interface, and structure, which was significant in the case of accuracy rate, *F*_(3.757, 157.774)_ = 3.068, *p* < 0.05, η^2^ = 0.068, but not for reaction time, *F*_(2.634, 110.641)_ = 1.439, *p* > 0.05, η^2^ = 0.033. Table 4 reports the results of the *post hoc* contrasts (Sidak) of estimated marginal means conducted to identify the specific differences that were significant.

**TABLE 4:** Pairwise comparison results for the three-way interaction in accuracy rate.

Structure	Interface	Intermediate vs. Advanced		Intermediate vs. Native		Advanced vs. Native	
		Mean difference	SE	Mean difference	SE	Mean difference	SE
Bare N	Internal	−15.45**	3.13	−13.06**	3.13	2.39	3.13
	External	−12.57**	3.12	−14.09**	3.12	−1.52	3.12
Cl-N	Internal	−18.92**	6.07	−38.84**	6.07	−19.53**	6.07
	External	−16.55**	4.14	−25.99**	4.14	−9.44	4.14
Num-Cl-N	Internal	−14.26**	3.83	−20.31**	3.83	−6.06	3.83
	External	−17.73**	4.33	−26.07**	4.33	−8.34	4.33

**p < 0.01.

Concerning the differences between the intermediate group and the other two groups, the pairwise comparison results reported in Table 4 were the same as those related to RQ1 and RQ2, i.e., the intermediate group performed significantly worse than the advanced and native groups regardless of syntactic structure and interface. However, the difference between the advanced group and the native group was more complex than the results associated with RQ1. The advanced group performed significantly worse than the native group at the intersection between the internal interface and the [Cl-N] structure, but no significant difference was found at other intersections. In other words, the advanced group has achieved nativelike attainment of the target expressions in most cases, except the internal interface of the [Cl-N] structure. In summary, to answer RQ3, the evidence above indicated that the heritage speakers’ acquisition of the target interface-regulated referential expressions was mediated by syntactic structure complexity.

### Discussion

To recapitulate, three hypotheses have been posed under this study in accordance with the IH: (1) the advanced adult heritage speakers can achieve target-like attainment of the target expressions at the internal interface but not at the external interface; (2) the heritage speakers’ performance at the syntax-semantics interface, but not at the syntax-discourse interface, is positively related to the heritage language proficiency level; and (3) the more complex the syntactic structure is, the more difficult for the heritage speakers to master the interface constraints on the use of the structure.

Hypothesis 1 is not confirmed by either accuracy data or reaction time data. Specifically, for accuracy, the advanced adult Chinese heritage speakers did not master either the syntax-semantics or syntax-discourse interface knowledge to a nativelike level, which suggests vulnerability of both internal and external interfaces. For reaction time, the advanced group exhibited nativelike performance at the syntax-discourse interface, hence not indicating a greater processing burden of the external interface knowledge. Likewise, Hypothesis 2 is not supported by accuracy and reaction time data, given the observation that the heritage speakers’ performance at both syntax-semantics and syntax-discourse interfaces could be improved—in terms of accuracy and reaction time—with the development of their Chinese proficiency.

The picture related to Hypothesis 3 is rather complicated. Upon taking into consideration the factor of syntactic structure complexity, the accuracy data revealed that the use of the [Cl-N] structure regulated at the internal interface, but not that at the external interface, presented prolonged difficulties for the advanced group; while the other two structures (i.e., bare Ns and [Num-Cl-N]) could be mastered by the advanced group to a nativelike level, irrespective of the interface type. This refuted Hypothesis 3 in two ways. First, Hypothesis 3 assumes that the advanced group would achieve nativelike attainment at the internal interface but not at the external interface, but our findings regarding [Cl-N] suggested the opposite. Second, Hypothesis 3 assumes that the more complex the syntactic structure is, the more difficult it is for the heritage speakers to acquire, according to which the [Num-Cl-N] is supposed to be the most challenging structure; nevertheless, the results showed that the syntactically less complex [Cl-N] was the most difficult one for the advanced group.

The above observations bring about several important implications to our understandings about the IH. First, the findings did not testify the claimed internal vs. external interface distinction regarding heritage language acquisition. Rather, it was revealed that the internal interface could also be a locus of non-convergence for even the advanced adult heritage speakers, as evidenced by the observation that the mean accuracy rate of the items related to the internal interface was significantly lower than that of the native group (cf. Table 3). This suggests that the type of interface should not be taken as a determinative factor for predicting the vulnerability of bilingual grammar knowledge.

Second, the present study did not lend support to a viewpoint underpinning the latest version of the IH, that is, the external interface imposes a greater processing burden for bilingual learners, which has been considered to be the fundamental reason for non-nativelike performance at the external interface (Sorace, 2011). In accordance with the reaction time data collected, no significant differences were observed between the mean reaction times the advanced group and the native group spent on the external interface items (cf. Table 3). This strongly indicates that the external interface should not necessarily be more taxing in processing.

Third, the intersection between interface (internal vs. external) and syntactic structure complexity points out a direction of inquiry worth future exploration. Under the present study, the use of the [Cl-N] structure regulated at the internal interface was found to be particularly difficult, even for the advanced group. Notice that this could be due to various possible reasons. For example, it could be partly explained in that the linguistic properties located at the internal interface might not be equivalently easy for learners, due to which some internal-interface properties could be more easily acquired to a nativelike level whereas others may not (cf. White, 2011). Along this line, the pattern regarding [Cl-N] could be considered in that the internal interface condition on the use of [Cl-N] happens to belong to the particularly difficult ones. Alternatively, this might be brought about by the fact that the learners did not receive sufficient or effective instruction on the use of [Cl-N], or that the input of this construction they had been exposed to was quantitatively/qualitatively insufficient. In fact, as has been pointed out in previous linguistic research, the appropriate use of [Cl-N] as an indefinite individual-denoting expression in Chinese would additionally be subject to complex genre- and prosody-related restrictions (Li and Feng, 2015). Since these restrictions are largely generalized under theoretical linguistic frameworks which Chinese language instructors may not be so familiar with, it is highly possible that the instructors themselves have not been fully aware of the using conditions of [Cl-N], hence the difficulty to provide precise descriptions and pedagogical explanations for this structure in class.

In addition to the theoretical implications, there are three pedagogical implications yielded by the present study. First, as both syntax-semantics and syntax-discourse grammar knowledge could possibly be a locus of learning difficulties for learners of Chinese, it is important for teacher educators and teachers to enhance their pedagogical awareness for precisely identifying and diagnosing the types of errors made by learners, and provide explicit, effective instruction on the observed challenging expressions. Second, upon the observation that it is not impossible for heritage speakers of Chinese to master the external interface grammar knowledge to a nativelike level, it is worthwhile exploring more innovative ways to teach discourse-related grammar rules (involving “variations” of language use) in class, which might be passed unnoticed by students. Last but not least, while linguistic research and language teaching have long been taken as two separate camps, the present study suggests that synergy between the two would be beneficial for language instructors *via* enriching their knowledge about linguistic subtleties, which can help to better inform pedagogy [see also Tao, 2016]. To realize such synergy, undoubtedly, more communication and cross-border collaboration are called for between linguists and teaching practitioners in the future (Gong et al., 2018, 2020).

### Conclusion

This paper tested the influential IH from the perspective of acquisition of Chinese as a heritage language. An internet-based AJT was administered to a total of 58 advanced and intermediate adult Chinese heritage speakers to examine their accuracy and real-time processing of referential nominal expressions regulated at the syntax-semantics and syntax-discourse interfaces. The target linguistic phenomena involved three nominal expressions in Chinese (i.e., Ns, [Cl-N], and [Num-Cl-N]) under four interface-regulated referential readings (i.e., type-denoting, quantity-denoting, indefinite individual-denoting, and definite individual-denoting). For accuracy, the results showed that (i) for bare Ns and [Num-Cl-N], regardless of the interface type, the advanced group mastered the target phenomena to a nativelike level, who significantly outperformed the intermediate group; (ii) for [Cl-N], the advanced group exhibited nativelike attainment at the syntax-discourse interface but not at the syntax-semantics interface, and performed significantly better than the intermediate group at both interfaces. For reaction time, no significant differences were reported between the advanced group and the native group regarding the learning of the target phenomena at either the syntax-semantics or the syntax-discourse interface, while the advanced group performed significantly better than the intermediate group, regardless of the interface type and the structure type. The present study adds new empirical evidence in heritage language acquisition to argue against the predictive power of the IH. The data suggest that the internal vs. external interface distinction cannot be taken as a reliable predictor for the possibility of nativelike performance in accuracy or real-time processing.

There are three main limitations of the present study. The first limitation is about the criterion adopted for dividing participants. We categorized the heritage speaker participants into advanced and intermediate groups based on their age of initial exposure to Chinese learning at school coupled with the participants’ self-rating on their overall Chinese proficiency. While the current division of the participant groups based on the age of onset of Chinese language learning was well validated by the participants’ self-evaluation of their Chinese proficiency, this measure may not be adequately rigorous and objective. Second, the present study did not look into the correlation, if any, between individual difference (ID) variables and the learners’ performance at the language interfaces, hence a lack of attention to possible compounding effects brought about by learner-related factors. Lastly, the Chinese heritage speaker participants were recruited from universities in mainland China. It is important for future research to compare findings in and outside of mainland China (Gong et al., 2018, 2020). We expect to further enrich the research in the future in three directions. First, for a more rigorous evaluation of the participants’ proficiency levels, in future research we may incorporate a separate Chinese proficiency test for dividing participant groups. Second, to obtain a holistic understanding about heritage language acquisition, there is a need to further take into consideration the heritage speakers’ family dialect background (e.g., Cantonese, Hakka), the dominate language of the country in which they were raised and grew up, and possible interactions in using heritage and societal languages in various contexts when examining heritage speakers’ mastery of interface knowledge in Mandarin Chinese. Third, the present experiment could be repeated with L2 Chinese learners in different educational contexts, and then the data of the L2 Chinese learners could be compared with the data collected under the present study to further explore whether there are any differences between L2 speakers and heritage speakers in acquiring bilingual grammars at interfaces. Pedagogically speaking, evidence of this study suggests that the use of certain interface-regulated expressions (e.g., [Cl-N]) may require more formal and contexualized instruction in heritage Chinese classrooms. With the experimental results showing the learnability of different types of interface knowledge for heritage speakers, the present study calls for more efforts to enhance Chinese language teachers’ linguistic awareness of the discourse-related grammar rules and their pedagogical awareness in teaching these rules in a way that could effectively cater for learner diversity.

## Data Availability Statement

The original contributions presented in the study are included in the article/supplementary material, further inquiries can be directed to the corresponding author.

## Ethics Statement

The study involving human participants was reviewed and approved by The Education University of Hong Kong. All the participants signed an informed consent form online to participate in this study.

## Author Contributions

All the authors listed have made a substantial, direct, and intellectual contribution to the work, and approved it for publication.

## Conflict of Interest

The authors declare that the research was conducted in the absence of any commercial or financial relationships that could be construed as a potential conflict of interest.

## Publisher’s Note

All claims expressed in this article are solely those of the authors and do not necessarily represent those of their affiliated organizations, or those of the publisher, the editors and the reviewers. Any product that may be evaluated in this article, or claim that may be made by its manufacturer, is not guaranteed or endorsed by the publisher.

## Footnotes

1 Take the word order of prenominal modifiers in the numeral classifier construction in Mandarin Chinese to illustrate interface phenomena. Generally, a modifier that denotes a stable, non-episodic property of the modified noun can only occur in between [Num-Cl] and the head noun but cannot precede the [Num-Cl] sequence in Mandarin Chinese, a restriction applied at the syntax-semantics interface, as shown in (i) below.

However, such a restriction can be overridden at the syntax-discourse interface: a modifier denoting a stable, non-episodic property of the modified can be allowed to appear in front of [Num-Cl] if the modifier is associated with a contrastive focus reading (Jin, 2020). As exemplified in (ii) below [adapted from Jin (2020)], when there are three long-haired girls that contextually receive a contrastive focus (i.e., contrasting with the two short-haired girls), the modifier *chang toufa de* “long-haired” can precede the [Num-Cl] sequence. (ii) Context: A and B just passed by five girls, three with long hairs and two with short hairs.

For an empirical study of adult L2 Chinese acquisition of the above word order phenomenon at the syntax-semantics and syntax-discourse interfaces, readers are referred to Jin and Ke (2021).

2 For the similarities and differences between L2 acquisition and heritage language acquisition, interested readers are referred to Lynch (2003) and Montrul (2010a,b, 2012), and the references therein.

3 Likewise, much less attention has been paid to L2 acquisition at interfaces in East Asian languages if compared with the research on L2 acquisition at interfaces in Romance, Germanic, and Slavic languages. Regarding language interfaces in L2 Chinese, specifically, the target phenomena touched upon in previous studies include *wh*-topicalization (Yuan and Dugarova, 2012; Dugarova, 2014), the *shi*…*de* cleft construction (Mai, 2013), *daodi* … *wh*-questions (Yuan, 2013), the overt pronoun *ta* “he/she” or a null element as an anaphora resolution (Zhao, 2014), word ordering of prenominal modifiers in numeral classifier sequences (Jin and Ke, 2021). Interested readers are referred to these studies for details.

4 Specific examples of each target expression under different referential meanings can be found in the Appendix.

5 The Chinese learners were invited to self-evaluate the level of Chinese proficiency along three 10-point Likert scales in listening, reading, and speaking: 0–totally not understand; 1–very poor; 2–poor; 3–fair; 4–slightly less than average; 5–average; 6–slightly more than average; 7–good; 8–very good; 9–excellent; and 10–perfect. The 10-point Likert scale follows Marian et al. (2007) design with our adjustments.

## Appendix: Sample Items of the Online Acceptability Judgment Task

I. Bare Ns

(a) Type-denoting (internal interface; acceptable reading)
  语境： A 和 B 在谈论水果。
  A：你最喜欢什么水果？
  B：我最喜欢香蕉。
(b) Quantity-denoting (internal interface; unacceptable reading)
  语境： A 和 B 在谈论做蛋糕。
  A：你做这个蛋糕放了多少糖？
  B：我放了白糖。
(c) Indefinite individual-denoting (external interface; acceptable reading)
  语境： A 和 B 在谈论早饭。
  A：你今天早饭做了什么吃？
  B：我煮了鸡蛋。
(d) Definite individual-denoting (external interface; acceptable reading)
  语境： B 新买了一双球鞋和一双皮鞋。
  A：这两双鞋贵吗？
  B：球鞋很便宜，皮鞋贵一点。

II. [Cl-N]

(a) Type-denoting (internal interface; unacceptable reading)
  语境： A 和 B 在谈论动物。
  A：你最怕什么动物？
  B：我最怕只猫。
(b) Quantity-denoting (internal interface; unacceptable reading)
  语境： A 和 B 在谈论借书。
  A：你昨天在图书馆借了多少书？
  B：我只借了本书。
(c) Indefinite individual-denoting (external interface; acceptable reading)
  语境： A 和 B 在讨论周末的安排。
  A：你上个周末做了什么？
  B：我看了部电影。
(d) Definite individual-denoting (external interface; unacceptable reading)
  语境：桌子上有一支钢笔和一支铅笔。
  A：这两支笔你想要哪一支？
  B：我想要支铅笔。

III. [Num-Cl-N]

(a) Type-denoting (internal interface; unacceptable reading)
  语境： A 和 B 在谈论花。
  A：你喜欢什么花？
  B：我喜欢一朵玫瑰花。
(b) Quantity-denoting (internal interface; acceptable reading)
  语境： B 参加了唱歌比赛。
  A：你比赛时一共唱了几首歌？
  B：我只唱了一首歌。
(c) Indefinite individual denoting (external interface; acceptable reading)
  语境： B 的样子看起来不太开心。
  A：发生什么事了？
  B：我打碎了一个花瓶。
(d) Definite individual-denoting (external interface; unacceptable reading)
  语境： A 在 B 的家里看到一只猫和一只狗。
  A：它们都是你买来的吗？
  B：不是，一只狗是朋友送的。
